# Development and Preliminary Application of Temperature Stress Test Machine for Cast-in-Place Inner Shaft Lining

**DOI:** 10.3390/ma16124351

**Published:** 2023-06-13

**Authors:** Chi Zhang, Shuaishuai Wang, Tao Zhang, Dahai Li, Hairui Chen

**Affiliations:** 1 State Key Laboratory for Geomechanics and Deep Underground Engineering, China University of Mining and Technology, Xuzhou 221116, China; ts19030195p31@cumt.edu.cn; 2 School of Transportation and Civil Engineering, Nantong University, Nantong 226019, China; zt0818@ntu.edu.cn; 3 China Petroleum Pipeline Engineering Co., Ltd., Langfang 065000, China; lidahai@cppmde.com; 4 Shanxi Zhengtong Coal Industry Co., Ltd., Xianyang 713600, China; chr518@126.com

**Keywords:** temperature stress test machine, concrete, hydration heat, shaft lining, degree of restraint

## Abstract

Over the past 20 years, as the depth and diameter of shaft lines increased in China, the cracking and water leakage of the inner walls of frozen shafts have become increasingly severe, resulting in significant safety threats and economic losses. Understanding the stress variation patterns of cast-in-place inner walls under the combined effects of temperature and constraint during construction is a prerequisite for evaluating the crack resistance performance of inner walls and preventing water leakage in frozen shafts. The temperature stress testing machine is an important instrument for studying the early-age crack resistance performance of concrete materials under the combined effects of temperature and constraint. However, existing testing machines have shortcomings in terms of applicable specimen cross-sectional shapes, temperature control methods for concrete structures, and axial loading capacity. In this paper, a novel temperature stress testing machine suitable for the inner wall structure shape, capable of simulating the hydration heat of the inner walls, was developed. Then, a reduced-scale model of the inner wall according to similarity criteria was manufactured indoors. Finally, preliminary investigations of the temperature, strain, and stress variations of the inner wall under 100% end constraint conditions were conducted by simulating the actual hydration heating and cooling process of the inner walls. Results show that the hydration heating and cooling process of the inner wall can be accurately simulated. After approximately 69 h of concrete casting, the accumulated relative displacement and strain of the end-constrained inner wall model were −244.2 mm and 187.8 με, respectively. The end constraint force of the model increased to a maximum value of 1.7 MPa and then rapidly unloaded, causing the model concrete to crack in tension. The temperature stress testing method presented in this paper provides a reference for scientifically formulating technical approaches to prevent cracking in cast-in-place concrete inner walls.

## 1. Introduction

Since 2000, deep soil layers, deep pore water-rich rock layers, and complex layers that have both of these have been commonly encountered while developing coal, iron, and other resources in China [1,2,3,4,5,6,7]. For these complex layers, the artificial freezing method is the most commonly used drilling method [8,9,10,11], accounting for over 90%. According to incomplete statistics, China has built over 150 vertical shafts using the freezing method in deep and complex layers in the past 20 years. The largest shaft net diameter *D* has reached 10.5 m (Beijiang Haizi Coal Mine Auxiliary Shaft, Nalunhe No. 2 Coal Mine Secondary Shaft), the largest frozen soil layer thickness *H*_s_ has reached 754.96 m (Wanfu Coal Mine Auxiliary Shaft, creating a world record), and the largest frozen bedrock depth *H*_d_ has reached 990 m (Gaojiappao Coal Mine West Huifeng Shaft, creating a world record).

**Figure 1:**
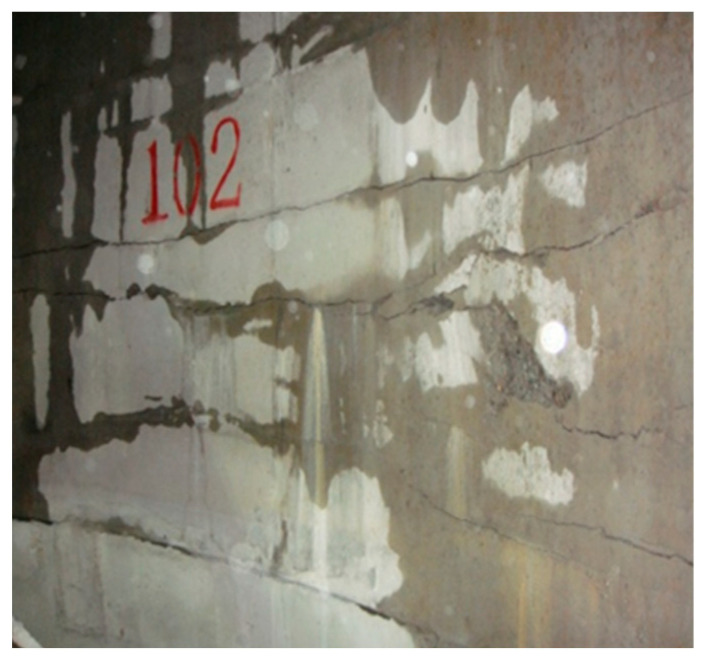
Leaking water in the Inner shaft lining.

However, these deep (freezing depth ≥ 400 m) and large (*D* ≥ 8 m) vertical shafts often suffered serious water leaks from the inner shaft lining after the frozen wall thawed, as shown in Figure 1 [12,13]. For example, the Auxiliary Shaft of Longgu Coal Mine (*D* = 7 m, *H*_s_ = 567.7 m, *H*_d_ = 650 m), the Auxiliary Shaft of Hetaoyu Coal Mine (*D* = 9 m, *H*_s_ = 214.6 m, *H*_d_ = 950 m), and the main shaft of Gaojiapu Coal Mine (*D* = 7.5 m, *H*_s_ = 26.5 m, *H*_d_ = 791 m) experienced leakages of 34 m^3^/h, 53 m^3^/h, and 74.2 m^3^/h, respectively, during construction, far exceeding the allowed values in the GB 50384-2016 specification published by Ministry of Housing and Urban-Rural Development of China [14].

The vertical shaft is the throat of a mine, and its safety is crucial to the entire mine. Severe leaks in the inner shaft lining not only significantly increase the cost of grouting and water control, extend the construction period of the mine, and cause severe economic losses but also pose a significant safety threat to the mine, which may trigger major malignant accidents, such as mud bursts and mine floods. From 1–8 January 2017, the vertical shaft of the Auxiliary Shaft of the already-in-production Longgu Coal Mine (*D* = 7 m, *H*_s_ = 567.7 m, *H*_d_ = 650 m) leaked at depths of 382 m and 418 m, and the total flow of water quickly skyrocketed from an initial 6~8 m^3^/h (containing approximately 5% mud and sand) to 105 m^3^/h (containing approximately 15% to 30% mud and sand), which was extremely dangerous. According to in-site test data, about 2000 m^3^ of mud and sand rushed into the vertical shaft before the rescue was successful. It can be seen that preventing leaks from the inner shaft lining of deep and frozen vertical shafts is important for ensuring the safety and production of the vertical shaft and the entire mine.

**Figure 2:**
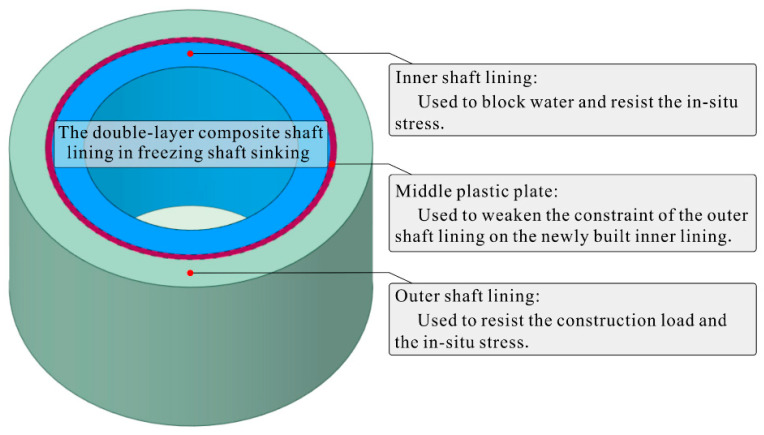
Schematic of the double-layer composite shaft lining structure in the freezing shaft sinking [18].

The above deep and large frozen vertical shaft commonly uses the double-layer reinforced concrete shaft lining structure with a sandwiched plastic plate (referred to as double-layer composite shaft lining), as shown in Figure 2, which is widely used globally [15,16]. Before 2000, the freezing depth of the Chinese double-layer composite shaft lining was generally less than 400 m, the net diameter of the vertical shaft was rarely more than 8 m, the thickness of the inner shaft lining was generally not more than 1 m, and the concrete strength grade was not higher than C55. Currently, the sandwiched plastic plate can effectively reduce the constraint of the outer shaft lining on the newly built inner shaft lining, leading to less temperature deformation cracks of the inner shaft lining concrete [17]. Actual surveys indicate that many near-horizontal circumferential cracks gradually appear on the inner edge of the inner shaft lining with increasing vertical shaft freezing depth and diameter, as shown in Figure 1. These cracks are the main leaky water channels of the vertical shaft, and such cracks appear even before the frozen wall thaws during construction. Theoretical analysis can exclude the possibility of inner shaft lining cracking from the points of shaft lining self-weight and formation water pressure. Preliminary judgment is that the cracking of the inner shaft lining concrete may be related to the large thickness of the inner shaft lining (up to 1~2 m) and the high-strength concrete (up to C60~C90). However, the exact failure mechanism of inner shaft lining concrete during construction is currently unclear, and there is no literature on the anti-cracking performance of cast-in-place inner shaft lining structure.

There are two dynamic processes in the freshly poured concrete: (1) the cementitious material quickly undergoes a hydration reaction, leading to an increase in concrete strength rapidly, and (2) the temperature change and self-shrinkage of the concrete cause volume shrinkage, resulting in tensile damage of the concrete [19,20,21]. Therefore, temperature change and constraint conditions are important factors affecting the concrete cracking of cast-in-place inner shaft lining during construction. The temperature change of cast-in-place inner shaft lining during construction includes two stages: (1) an initial stage where the temperature rises to the maximum temperature with the concrete hydration reaction, and (2) a later stage where the temperature gradually decreases with the influence of the frozen walls and air temperature in the vertical shaft [22]. Therefore, it is necessary to study the stress variation characteristic of the inner wall during construction under the combined effects of temperature and constraint to accurately evaluate the cracking resistance of the inner wall. For the inner shaft lining within a certain height range, the external constraints include the upper- and lower-end constraints and the outer circumferential surface constraint.

Existing studies have shown that the Temperature Stress Testing Machine (TSTM) is a powerful instrument for studying the early-age anti-cracking of concrete under the combined effects of temperature and constraint. TSTMs can accurately control the constraint conditions of the cast-in-place concrete specimens and simulate the tensile damage and cracking process of concrete [23,24,25,26,27,28,29,30]. Riding et al. [31] used a rigid cracking frame to measure the uniaxial stress of concrete containing different fly ashes under restrained conditions, and the results showed that fly ashes reduced the cracking risk because of the decrease in the heat of hydration of the cementitious materials. Kovler [32] presented a modified uniaxial restrained shrinkage test for early-age concrete to make it possible to resolve creep strain from shrinkage strain. Shen et al. [33] used a TSTM to investigate the influence of hooked-end steel fiber and thermal treatment temperature on the early-age tensile creep of concrete under a constant tensile loading, and the result showed that the early-age autogenous shrinkage of concrete decreased with an increase of the content of hooked-end SF and increased with an increase of the thermal treatment temperature. However, studies regarding the restrained thermal and autogenous shrinkage deformations of the shaft structure remain insufficient. Additionally, most of the TSTMs with limited loading capacities are applied to low-strength concrete, which is inadequate for the shaft walls casted by high-strength concrete.

Considering the limitations of existing testing machines in terms of applicable specimen cross-sectional shapes, temperature control methods for cast-in-place concrete structures, and axial loading capacity, this study aims to develop a novel TSTM suitable for the shaft structure. With a similar simulation test, a preliminary investigation of the temperature, strain, and stress variations of the cast-in-place inner shaft lining under full end constraint degree (*K_r_* = 100%) during the processes of early-age hydration heat and later cooling is studied. The constraint factor *K_r_* refers to the degree of the end constraint of relative horizontal displacement,

(1) $$K_{r}=\left(X_{f}-X_{c}\right)/X_{f}$$

where *X_f_* is the relative horizontal displacement of the inner shaft lining scaled model under unrestrained conditions at the end, and *X_c_* is the allowed relative horizontal displacement of the scaled model. Therefore, *K_r_* = 0 means that the end of the inner shaft lining scaled model can freely expand and contract in the horizontal direction, while *K_r_* = 100% means that the allowed relative horizontal displacement of the left and right ends of the inner shaft lining scaled model is 0.

## 2. Test Methodology

### 2.1. Development of the Testing Machine

**Figure 3:**
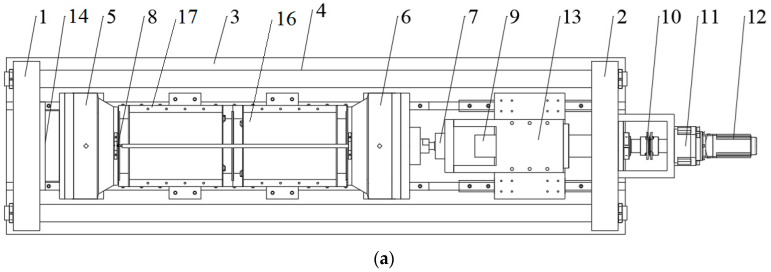

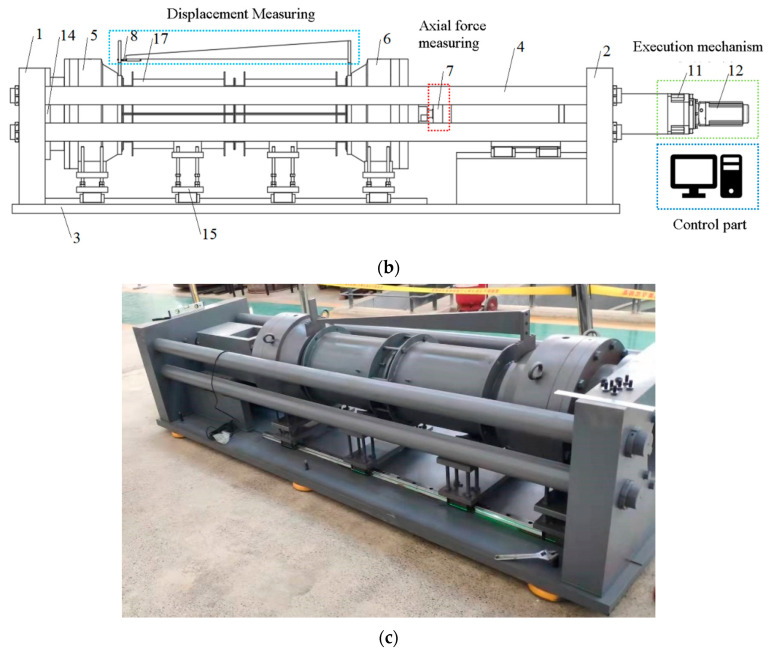
Schematic diagrams of the TSTM (**a**) top view; (**b**) front view; (**c**) physical map. 1—End bracket; 2—Supporting bracket; 3—Base plate; 4—Pull rod; 5—Fixed end clamp; 6—Movable end clamp; 7—Axial force sensor; 8—Displacement sensor; 9—Precision screw; 10—Coupling; 11—planetary reducer; 12—Stepping motor; 13—Guide component; 14—Connecting flange; 15—Slide rail support system; 16—Inner shaft lining scaled model; 17—Inner shaft lining template.

A novel TSTM was developed to cover limitations in the loading capacity and structure style of conventional TSTMs. The TSTM consists of a restrained frame system, a slidable support system, and a measurement control system, as shown in Figure 3. The restrained frame system is mainly formed by a rigid frame, a fixed end, and a moveable end, which are used to apply the displacement constraint from 0% to 100%. The slidable support system is used to support the weight from above and allows horizontal displacement due to temperature dilation. The measurement control system comprises a displacement control unit and several displacement and pressure sensors. The horizontal loading speed driven by the combination of a stepping motor and a planetary reducer (the reduction ratio is 50) ranges from 0.01 mm/min to 20 mm/min.

In actual engineering, the net diameter of the inner wall is designed from 8 m to 10 m, and its thickness is designed from 1 m to 2 m. To achieve similarity in the model test, it is necessary for the scale test to simulate the geometry. In this study, the geometric scale ratio is taken as 40, and the ratio of height to external diameter is taken as 4. From this, the net diameter of the simulated inner wall ranges from 200 mm to 250 mm, the external diameter of the simulated inner wall ranges from 225 mm to 300 mm, and the height of the simulated inner wall ranges from 900 mm to 1200 mm. Thus, the required minimum testing space is 1200 mm × 300 mm × 250 mm (length × width × height). Given the extra working space, the final testing space is 2500 mm × 650 mm × 650 mm. The net diameter and the external diameter of the simulated inner wall are 200 mm and 300 mm, respectively, from which the loading capacity F of the TSTM can be calculated.

(2) $$F=\pi f_{t}\left(300^{2}-200^{2}\right)/4$$

where *f_t_* refers to the tensile strength of high-strength concrete C100, which is common in deep shafts. *f_t_* equals 7.32 MPa for the steel fiber-reinforced concrete [34]. The ultimate loading capacity of the TSTM is set to 300 kN, which is slightly larger than the theoretical value. Table 1 lists the sizes of inner walls in actual engineering and model test.

### 2.2. Temperature Field Measurement Method of the Simulated Inner Wall

**Figure 4:**
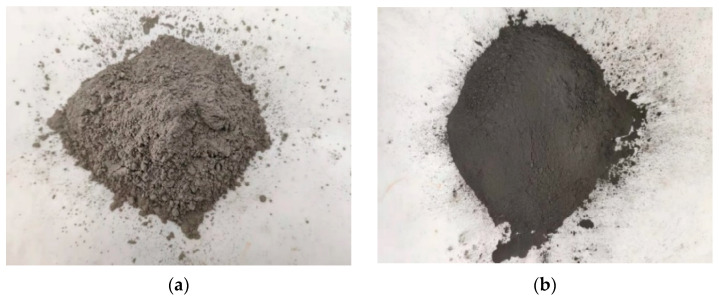

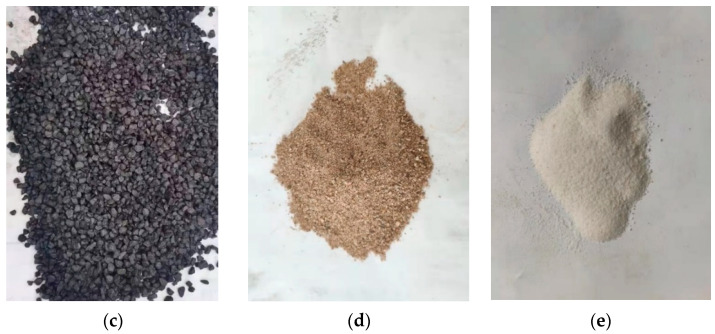
Illustrations of the raw materials of concrete: (**a**) cement; (**b**) iron slag; (**c**) basalt-based gravel ranging from 5 mm to 10 mm; (**d**) silica-based river sand; (**e**) water reducer.

The cubic compression strength of the conductive concrete (60 MPa) at 28 days was used in this study. A water-to-cement ratio of 0.31 was utilized for the concrete mixture. Conductive concrete reinforced with iron slag has been investigated extensively [35], and the concrete mixture proportion is listed in Table 2. P.O 52.5 cement was used, and its chemical composition is shown in Table 3. The coarse aggregate was basalt-based gravel with a size ranging from 5 mm to 10 mm, and the fine aggregate was common silica-based river sand. Figure 4 illustrates the raw materials of concrete. Generally, the internal temperature of the concrete is controlled through external heat conduction, which fails to simulate the internal hydration heat of the cast-in-place scaled model [23,36]. Research has shown that the problem of insufficient hydration heat of the cast-in-place concrete scaled model due to the reduction of the prototype structure size can be solved using the electrothermal effect [37]. The conductive concrete generates electrothermal energy under the action of direct current. The voltage of the direct current is adjusted according to the real-time temperature of the inner shaft lining scaled model to ensure that the temperature rise curve of the scaled model is consistent with the engineering prototype during the concrete hydration process.

### 2.3. Testing Procedure

**Figure 5:**
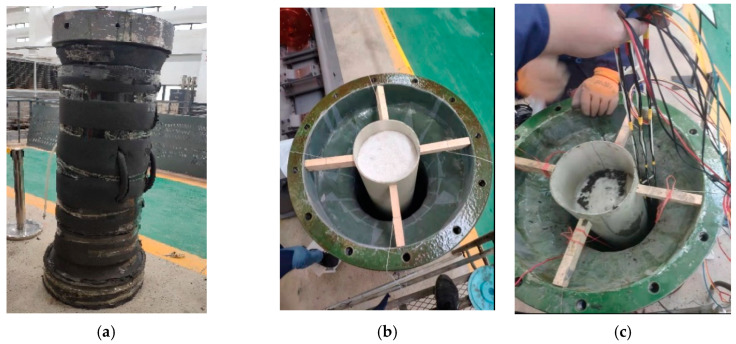

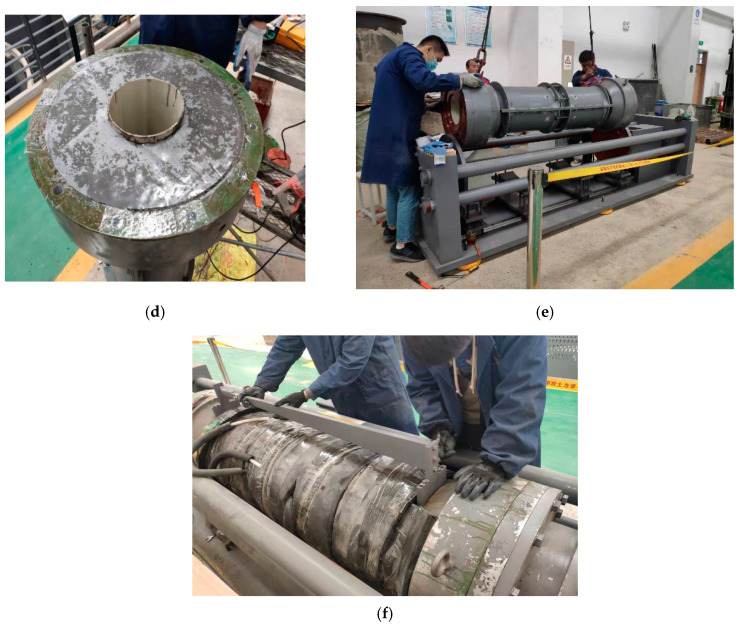
Testing procedure: (**a**) wrapping the heat preservation cotton outside the framework; (**b**) fixing the polypropylene pipe in the center of the concrete framework; (**c**) burying the thermistors and fiber optic sensors in the inner wall; (**d**) pouring concrete; (**e**) lifting the framework into the support system; (**f**) placing the concrete on the TSTM.

The TSTM testing procedure is shown in Figure 5. First, the concrete framework was assembled with the fixed and moveable ends into a whole and placing them vertically. The concrete framework was enclosed by heat preservation cotton, as shown in Figure 5a. Then, a layer of polyethylene film was applied to the inside of the concrete framework to simulate the sandwiched polyethylene plate in the double-layered composite shaft, and a polypropylene pipe was fixed in the center of the concrete framework, as shown in Figure 5b. Finally, the concrete of the inner wall was poured while burying the thermistors and fiber optic sensors, as shown in Figure 5c,d. The poured concrete was placed horizontally and was fixed at both ends on the TSTM, and later, the displacement and stress sensors were installed, as shown in Figure 5e. The test came to an end when the concrete temperature dropped to a minimum or the concrete cracked during the cooling process. 

### 2.4. Sensor Layout

**Figure 6:**
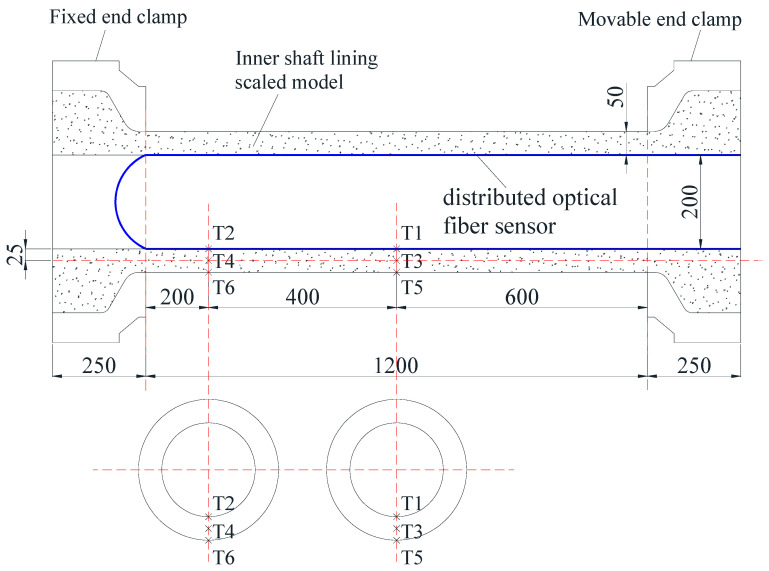
Layout of the thermistors and fiber optic sensors in the scaled model (unit: mm).

Figure 6 shows the layout of the thermistors and fiber optic sensors in the scaled model. The thermistors (T1~T6), buried along the radial direction of the concrete, were used to monitor the temperature variation. The fiber optic sensors (blue line in Figure 6), placed along the inner edge of the concrete, were used to monitor the concrete strain. One monitoring point was set up at 1 cm intervals along the length of the fiber optic.

## 3. Results

### 3.1. Temperature Field of the Inner Shaft Lining Scaled Model

**Figure 7:**
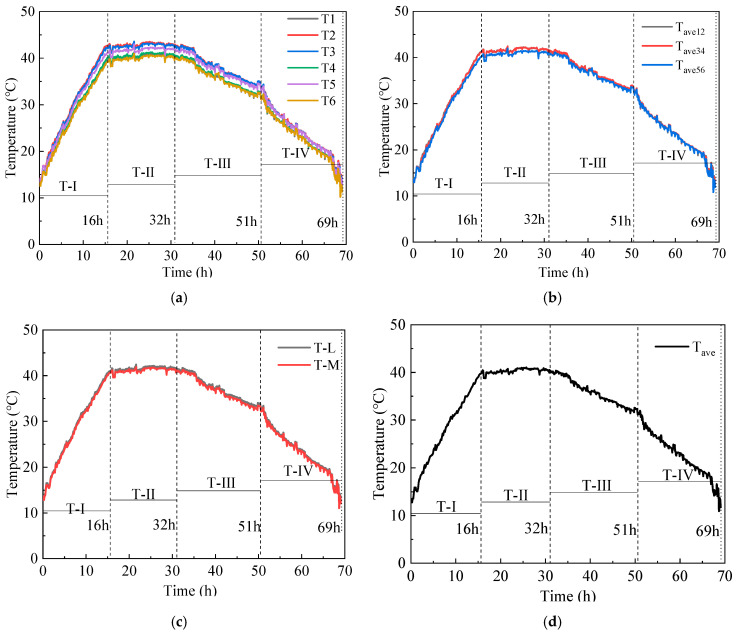
Temperature variation of the inner shaft lining scaled model (**a**) temperature at each temperature measurement point T1~T6; (**b**) average temperature at the scaled model inner edge T_ave12_, middle T_ave34_, and outer edge T_ave56_; (**c**) average temperature at the fixed end cross-section T-L and middle cross-section T-M; (**d**) average temperature T_ave_ for T1~T6.

Table 4 and Figure 7 show the temperature results and temperature variation of the shaft scaled model. The concrete entry temperature is 12.5 °C, as shown in Figure 7a. The results show temperature deviations between T1 and T2. For example, the deviation values are 0.60 °C, 2.60 °C, 2.61 °C, 2.54 °C, and 1.41 °C at the moments of 0 h, 16 h, 32 h, 51 h, and 69 h. The deviation values as a percentage of T2 are 4.5%, 6.0%, 6.2%, 7.5%, and 9.7%, respectively. The deviation values between T3 and T4 are 0.50 °C, 2.00 °C, 2.15 °C, 2.10 °C, and 1.23 °C, and the deviation values as a percentage of T4 are 3.9%, 4.9%, 5.4%, 6.7%, and 9.4%, respectively. The deviation values between T5 and T6 are 0.90 °C, 1.90 °C, 1.87 °C, 1.78 °C, and 1.25 °C, and the deviation values as a percentage of T6 are 7.2%, 4.8%, 4.7%, 5.7%, and 9.9%, respectively. The results show that the concrete is a non-homogeneous material, and the iron slag mixed with similar material in the conductive concrete could not be homogeneous. Therefore, the shaft model cannot be heated uniformly under the electrical heat effect.

Figure 7b shows the variation of the average temperatures of T_ave12_, T_ave34_, and T_ave56_, respectively. The maximum difference between T_ave12_ and T_ave34_ is only 0.2 °C. The temperature values of T_ave12_ and T_ave34_ are slightly higher than that of T_ave56_. For example, the temperature values of T_ave34_ are higher than T_ave56_ by about 0.0 °C, 1.0 °C, 0.7 °C, 0.3 °C, and 0.4 °C at the moments of 0 h, 16 h, 32 h, 51 h, and 69 h, respectively. This is consistent with the pattern of a large number of field measurements, i.e., the highest temperature of the cast-in-place inner wall occurs in the middle of the inner wall off the inner edge.

Figure 7c shows the variation of the average temperatures of the fixed end section (T-L) and the central section (T-M) of the shaft model. The maximum difference between T-L and T-M is 0.5 °C, indicating that the temperature is relatively uniformly distributed in the height direction of the shaft model.

Figure 7d shows the variation in the average temperature for all monitoring points. The curve can be divided into four stages: rapid heating stage (T-I), steady temperature stage (T-II), slow cooling stage (T-III), and rapid cooling stage (T-IV). In stage T-I (0~16 h), the temperature of the shaft model rises rapidly from the initial temperature of 13.0 °C to 41.4 °C with an average temperature rising rate of 1.78 °C/h, which is attributed to the excellent conductivity under the action of 300 V direct current. In stage T-II (16~32 h), the shaft model reaches thermal equilibrium with the ambient temperature of 5 °C, and the temperature smoothly maintains at 39.8~40.5 °C. In stage T-III (32~51 h), the concrete strength continues to grow, and the electrical conductivity further decreases. The shaft model slowly cools down with an average cooling rate of 0.45 °C/h. In stage T-IV (51~69 h), the shaft model rapidly cools with an average cooling rate of 1.01 °C/h due to the low-temperature brine.

### 3.2. Cumulative Strain

**Figure 8:**
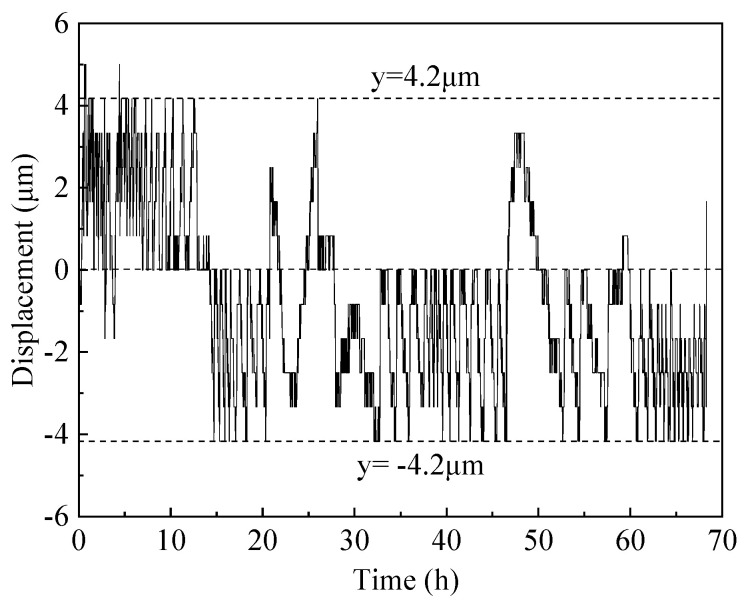
Real-time relative displacement variation between both ends of the scaled model.

The end restraint degree *K_r_* of the inner shaft lining scaled model in this test group is 100%, meaning that the allowed relative horizontal displacement of the two ends *X_c_* is 0, and the displacement threshold *X_t_* is ±5 μm. Therefore, the real-time relative displacement values of the two ends of the model should be between −5 μm and 5 μm. In Figure 8, the real-time relative displacement values of the model end measured are mostly between −4.2 μm and 4.2 μm.

**Figure 9:**
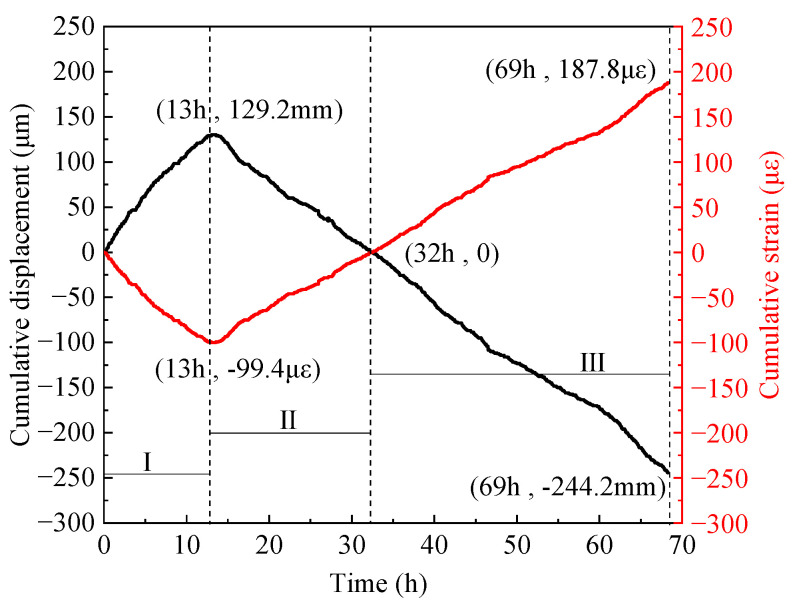
Cumulative displacement and strain variations of the scaled model.

Figure 9 shows the cumulative relative displacement of the model end, which is the accumulated value of the real-time relative horizontal displacement values in Figure 8. The change law of the cumulative relative displacement and cumulative strain of the model end can be divided into three stages: (1) the electrothermal effect of the cast-in-place conductive concrete similar material causes the inner shaft lining scaled model to undergo thermal expansion and temperature deformation from 0 to 13 h. The temperature deformation is superimposed with the self-shrinkage generated by the concrete material during the hardening process, forming the volume deformation of the inner shaft lining scaled model. This is manifested in the volume deformation of the scaled model in the height direction of the inner shaft lining; that is, the cumulative relative displacement of the model end increases to the maximum value of 129.2 mm at 13 h. The cumulative relative displacement generated by the thermal expansion deformation of the model end is compressed, resulting in compressive strain in the height direction of the scaled model. Correspondingly, the cumulative strain of the inner shaft lining scaled model decreases to the maximum compressive strain of −99.4 με at 13 h; (2) the average temperature T_ave_ of the inner shaft lining scaled model remains unchanged at 39.8 °C to 40.5 °C, as shown in Figure 7, and the temperature deformation of the scaled model is not significant from 13 to 32 h. The volume deformation of the inner shaft lining scaled model in the height direction is mainly composed of self-shrinkage of the concrete material. The cumulative relative displacement of the model end gradually decreases from the maximum value of 129.2 mm and decreases to 0 at 32 h when the model height is restored to 1200 mm. Correspondingly, the cumulative strain of the inner shaft lining scaled model increases from −99.4 με to 0; (3) the average temperature T_ave_ of the inner shaft lining scaled model is in the slow and rapid cooling stage, as shown in Figure 7, and the temperature deformation of the scaled model is significant from 32 to 69 h. The volume deformation of the inner shaft lining scaled model in the height direction is mainly caused by temperature shrinkage deformation. The cumulative relative displacement of the model end gradually decreases to −244.2 mm at 69 h. Correspondingly, the inner shaft lining the scaled model changes from a compressed state to a tensile state, and the cumulative strain of the scaled model gradually increases to 187.8 με at 69 h. 

### 3.3. Constraint Stress

**Figure 10:**
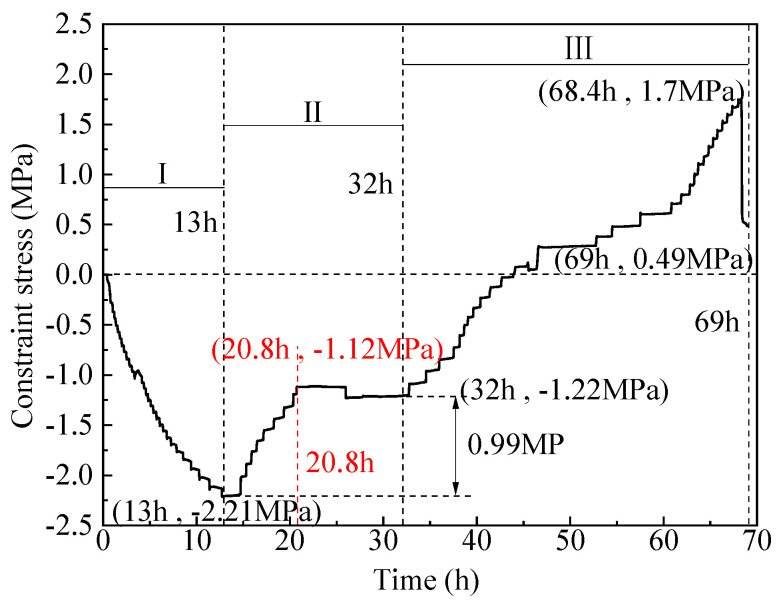
Constraint stress variation of the inner shaft lining scaled model.

Figure 10 shows the constrained stress of the inner shaft lining scaled model. The constrained stress is a concrete internal stress generated by the model volume deformation constrained in the vertical direction. The change in the constrained stress of the inner shaft lining scaled model can be divided into three stages: (1) the cumulative relative displacement of the model end increases to a maximum value of 129.2 mm at 13 h, as shown in Figure 9. Correspondingly, the constrained stress of the inner shaft lining scaled model decreases to a maximum compressive stress of −2.21 MPa at 13 h; (2) the cumulative relative displacement of the model end gradually decreases from the maximum value of 129.2 mm and decreases to 0 at 32 h. Correspondingly, the constrained stress of the inner shaft lining scaled model increases to −1.22 MPa at 32 h; (3) the cumulative relative displacement of the model end gradually decreases from 0 and decreases to −244.2 mm at 69 h, as shown in Figure 9. To achieve a constraint degree of *K_r_* = 100% for the model end, the cumulative relative displacement generated by the temperature deformation at the end should be stretched, and the constrained tensile stress at the model end gradually increases. The constrained tensile stress reaches its maximum value of 1.7 MPa at 68.4 h. Then, the constrained stress of the inner shaft lining scaled model rapidly unloads until the test completes.

**Figure 11:**
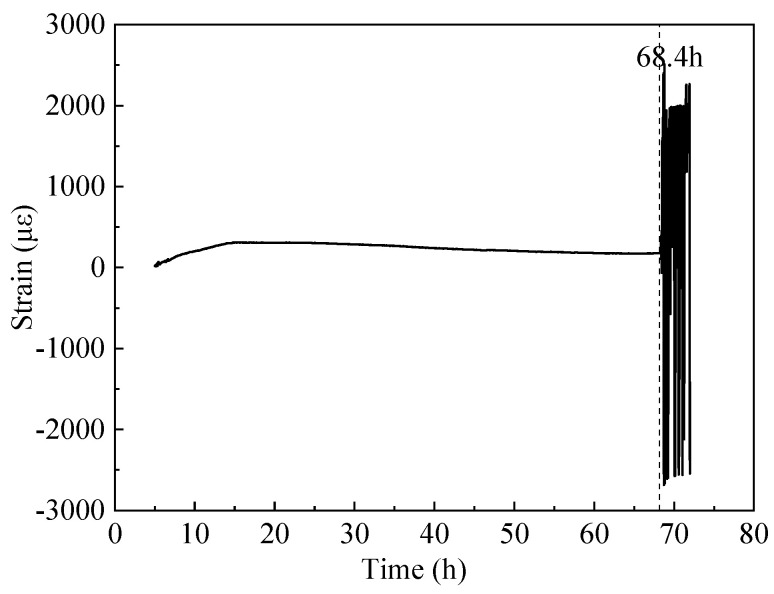
Cracking time of the inner shaft lining scaled model.

**Figure 12:**
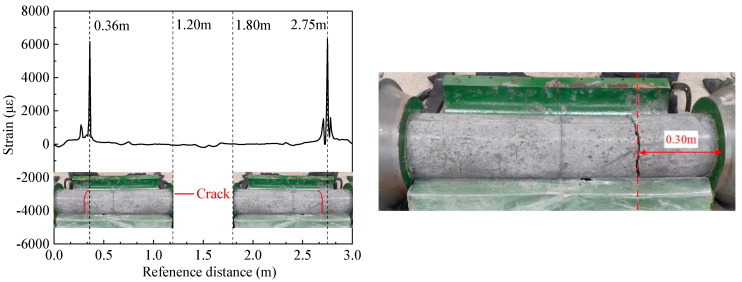
Cracking location of the inner shaft lining scaled model.

Figure 11 and Figure 12 show that the distributed fiber optic strain measurement points on the inner shaft lining scaled model exhibit a sudden increase in tensile strain and anomalous fluctuations at distances of 0.25 m and 0.36 m from the moveable end. The anomalous tensile strain values of around 6161 με to 6320 με far exceed the ultimate tensile strain of concrete, indicating that the inner shaft lining scaled model concrete near the moveable end clamp is under tensile cracking. After removing the model template when completing the test, it was found that the actual location where the model concrete cracked was approx. 0.30 m away from the moveable end clamp of the scaled model.

## 4. Conclusions

This paper developed a novel temperature stress test machine suitable for the inner wall structure shape, capable of simulating the hydration heat of the inner walls to simulate the internal hydration heating and cooling process of cast-in-place inner shaft lining. The machine accurately controls the 100% end constraint condition of the inner shaft lining scaled model for the first time. The salient conclusions are summarized as follows:

(1) The average temperature at the inner edge of the inner shaft lining scaled model is close to the average temperature in the middle, and both are slightly higher than the average temperature at the outer edge of the model by 0.3 °C to 1.0 °C. The temperature distribution in the height direction of the inner shaft lining scaled model is relatively uniform, with a maximum difference of 0.5 °C;
(2) The average temperature variation can be divided into four stages: rapid heating stage (T-I), steady temperature stage (T-II), slow cooling stage (T-III), and rapid cooling stage (T-IV). In stage T-I (0~16 h), the temperature of the shaft model rises rapidly from the initial temperature of 13.0 °C to 41.4 °C with an average temperature rising rate of 1.78 °C/h, which is attributed to the excellent conductivity under the action of 300 V direct current. In stage T-II (16~32 h), the shaft model reaches thermal equilibrium with the ambient temperature of 5 °C, and the temperature smoothly maintains at 39.8~40.5 °C. In stage T-III (32~51 h), the concrete strength continues to grow, and the electrical conductivity further decreases. The shaft model slowly cools down with an average cooling rate of 0.45 °C/h. In stage T-IV (51~69 h), the shaft model rapidly cools with an average cooling rate of 1.01 °C/h due to the low-temperature brine;
(3) The trends in the cumulative relative displacement and cumulative strain at the end of the inner shaft lining scaled model can be divided into three stages. From 0 to 13 h, the cumulative relative displacement at the model end increases to a maximum value of 129.2 mm at 13 h, and the cumulative strain of the inner shaft lining scaled model decreases to a maximum compressive strain of −99.4 με at 13 h. From 13 to 32 h, the cumulative relative displacement of the model gradually decreases and decreases to 0 at 32 h when the model height is restored to 1200 mm, and the cumulative strain of the inner shaft lining scaled model increases from −99.4 με to 0. From 32 to 69 h, the cumulative relative displacement of the model gradually decreases and decreases to −244.2 mm at 69 h. The scaled model changes from a compressed state to a tensile state, and the cumulative strain of the model gradually increases to 187.8 με at 69 h;
(4) The change in the constrained stress of the inner shaft lining scaled model can be divided into three stages. From 0 to 13 h, the constrained stress of the scaled model decreases to a maximum compressive stress of −2.21 MPa at 13 h. From 13 to 32 h, the constrained compressive stress at the model end gradually transitions to tensile stress, and the constrained stress of the scaled model increases to −1.22 MPa at 32 h. From 32 to 69 h, the constrained tensile stress at the model end gradually increases, and the constrained tensile stress increases to a maximum tensile stress of 1.7 MPa at 68.4 h. Then, the constrained stress of the scaled model rapidly unloads until the test ends at 69 h;
(5) It is feasible to determine the location and time of cracking in the scaled model by judging whether the tensile strain values at each measurement point of the distributed fiber optic sensor exceed the ultimate tensile strain of concrete. The abnormal points of the tensile strain value of the distributed fiber optic sensor are located at a distance of 0.25 m and 0.36 m from the movable end clamp. A certain concrete strain value near the crack of the scaled model showed a sharp increase in tensile strain at 68.4 h.

## Figures and Tables

**Table 1:** Size comparison of the engineering prototype and similar model.

Type	Geometric Scale Ratio	Net Diameter of Inner Wall (mm)	Thickness of Inner Wall (mm)	Thickness of Plastic Plate (mm)	Height of Inner Wall (mm)
Engineering prototype	1	8000	2000	3	—
Similar model	40	200	50	0.075	1200

**Table 2:** Concrete mixture proportion of simulated inner wall.

Water (kg)	Cement (kg)	Iron Slag (kg)	Fine Aggregate (kg)	Coarse Aggregate (kg)	Water Reducer (kg)
189	359	251	527	1173	1.22

**Table 3:** Chemical composition of cement.

SiO_2_	Al_2_O_3_	Fe_2_O_3_	CaO	MgO	SO_3_
20.83	4.74	2.91	61.26	3.29	2.30

**Table 4:** Details of the temperature of the scaled model.

Time/h	T1 /°C	T2 /°C	T3 /°C	T4 /°C	T5 /°C	T6 /°C	T_ave-12_ /°C	T_ave-34_ /°C	T_ave-56_ /°C	T-L /°C	T-M /°C	T_ave_ /°C
0	12.7	13.3	13.2	12.7	13.4	12.5	13.0	13.0	13.0	12.8	13.1	13.0
16	40.5	43.1	42.7	40.7	41.7	39.8	41.8	41.7	40.8	41.2	41.6	41.4
32	39.8	42.4	42.3	40.2	41.4	39.6	41.1	41.2	40.5	40.7	41.2	40.9
51	31.3	33.8	33.7	31.6	33.3	31.5	32.5	32.7	32.4	32.3	32.8	32.5
69	13.1	14.5	14.3	13.1	13.9	12.6	13.8	13.7	13.3	13.4	13.7	13.6

Note: T_ave12_ = (T1 + T2)/2; T_ave-4_ = (T3 + T4)/2; T_ave56_ = (T5 + T6)/2; T-L = (T2 + T4 + T6)/3; T-M = (T1 + T3 + T5)/3; T_ave_ = (T1 + T2 + T3 + T4 + T5 + T6)/6.

## Data Availability

Not applicable.
